# Supplementary material for: Towards explainable interaction prediction: Embedding biological hierarchies into hyperbolic interaction space
Source: PLoS One. 2024 Mar 21;19(3):e0300906. doi: 10.1371/journal.pone.0300906 (PMC10956837; doi:10.1371/journal.pone.0300906)
Supplement: Attachment — Submitted filename: response.pdf [file pone.0300906.s001.pdf]

Authors' Response to Reviews of

Towards explainable interaction prediction: Embedding biological hierarchies into hyperbolic interaction space

Domonkos Pogány, Péter Antal
PLoS ONE,

The authors would like to thank the editor and reviewers for their time and effort dedicated to providing constructive comments that have helped improve the quality of this manuscript. The manuscript has been thoroughly revised according to the reviewers' insightful comments and questions. In the marked-up copy of our manuscript (including the figures), we highlighted the changes made to the original version with the following notation: ~~previous~~revised. Please see our responses below, where page numbers refer to the marked-up manuscript file with tracked changes.

RC/Q: ***Reviewers' Comment/Question***, AR: Authors' Response, ☐ Manuscript Text

1. Reviewer #1

1.1. More diverse and complex datasets

RQ: ***"How does the model perform when subjected to more diverse and complex datasets beyond KIBA and NURA?"***

AR: Thank you for raising this important point.

While we primarily focused on the KIBA and NURA datasets in our study, we have previously tested similar model architectures without hierarchy regularization on larger datasets such as ChEMBL, BindingDB, and DrugBank, where the predictive performance remained consistent. However, for evaluating the impact of regularization, datasets with hierarchical information are essential. We acknowledge the need for more diverse and complex datasets beyond KIBA and NURA, but unfortunately, suitable options with labeled hierarchies are still limited. We did not find a dataset with known hierarchies larger than the KIBA. This information was indeed not provided in the manuscript, so we modified a paragraph in the *Methods/Datasets/Drug-target interactions* subsection (page 12) in the following way:

> One of them has *protein kinases* as targets, called the *Kinase Inhibitor BioActivity* (KIBA) [49] dataset, which is, to the best of our knowledge, one of the largest DTI datasets with rich, labeled hierarchical information.

Besides KIBA and NURA, we also tested the method on another smaller dataset with a different type of hierarchy and obtained similar results (see also in RQ 1.3.). We also acknowledged the challenge of the need for more abundant complex datasets with labeled hierarchies. The corresponding modifications are found in the *Discussion and conclusions* section (pages 29-30):

...
With these adjustments, we ran the model on a subset of the ChEMBL [67] dataset introduced in our previous study [18]. Utilizing the MeSH and ATC as prior hierarchies, we obtained similar results, achieving a high EDP score without significantly impacting predictive performance.
...
Another ...challenge to be addressed is the limited availability of public data containing abundant, labeled hierarchies, leading to the need for diverse DTI benchmarks and datasets focusing on biological hierarchies.

1.2. Other embedding techniques

RQ: ***"Are there other embedding techniques that were considered but not included in this study, and why?"***

AR: We appreciate the opportunity to expand on the embedding techniques considered for our study.

We have updated the *Methods/Datasets/Input representations* subsection to include additional information on techniques that were not previously mentioned (pages 12-13):

When exploring various molecule representations such as the Morgan fingerprint, including one-hot and categorical inputs without prior information, Morgan fingerprints with various lengths, SMILES descriptor, and the 300-dimensional, pre-trained *Mol2vec* embeddings [51], the latter proved most efficient and achieved superior performance.
...
For protein embeddings, we examined several pre-trained models on utilizing amino acid sequences [52]. Among these, *ProtVec* [53], employing which employs the Word2vec concept on protein sequences, emerged as a standard choice, and is commonly paired with Mol2vec [15] . While , emerged as a standard choice. While two of the other pre-trained methods, namely *CPCProt* [54] slightly improved [54] and *ProtTrans*[55], also seemed promising, with the former slightly improving predictive performance and *ProtTrans* [55] exhibited the latter exhibiting better hierarchy preservation, ProtVec offered a balanced trade-off between the two considerations. these considerations. Additionally, we explored alternative approaches, such as using one-hot and categorical inputs, as well as employing bag-of-words representations with k-mers of various lengths in the amino sequences. We also experimented with different dimensionality reduction techniques on the input representations and the combination of different embeddings, utilizing appropriate multimodal encoders. However, none of the methods listed above managed to outperform the pre-trained embeddings. Consequently, we selected opted for the 300-dimensional ProtVec protein embeddings and used them throughout our study.

Besides, there are other tested but not mentioned underperforming pre-trained protein embeddings, for example, SeqVec, ESM, and ESM1b. There are also other representations that were considered but not mentioned, such as utilizing raw amino sequences, SMILES molecule representations with a CNN or transformer encoder, or molecular graphs paired with a GNN encoder. We have worked with these techniques in our previous works. However, without pre-training, the models required to harness the information of these representations are usually too complex for the used medium-sized datasets. In our current study, we tried to keep the representations and the model simple to access only the effect of the hierarchy regularization and the chosen manifold. Additionally, there is another interesting input modality we started experimenting with only after we submitted the previous manuscript. These are the differentially expressed gene signatures, which we found suitable for hyperbolic models. These results were since published in another conference paper titled *Hyperbolic Drug-Target Interaction Prediction Utilizing Differential Expression*

Signatures. More information on this work can be found in the preliminary version of the proceedings: *https://www.mit.bme.hu/eng/kutatas/miniszimpozium/proceedings* (31st Minisymposium).

1.3. Different types of biological hierarchies

RQ: ***"How does the model's performance vary with different types of biological hierarchies?"***

AR: Thank you again for pointing this aspect out.

As mentioned, we also tested our method on a ChEMBL subset with MeSH and ATC hierarchies. In the revised version, we provided a paragraph in the *Discussion and conclusions"* section, including a brief overview of the results (page 29):

> The regularization can be extended to handle hierarchies where the internal nodes are represented as well. For example, when dealing with drug repositioning datasets where diseases serve as targets, the *Medical Subject Headings* (MeSH) [66] can be employed instead of protein hierarchies. Unlike protein hierarchies, MeSH incorporates targets not only at the leaves but also within internal nodes, i.e., it is possible that one target is a descendant of another. The only modification needed in our method is to replace the lowest common ancestor distance with the number of hops in the shortest path between two diseases. With these adjustments, we ran the model on a subset of the ChEMBL [67] dataset introduced in our previous study [18]. Utilizing the MeSH and ATC as prior hierarchies, we obtained similar results, achieving a high EDP score without significantly impacting predictive performance. Our experiences indicate the robustness of the regularization method across various hierarchy types. Including the NR with a regular tree structure, the kinase hierarchy with the other and atypical groups treated differently, the ATC with leaves belonging simultaneously to multiple labels in different hierarchy levels, and the MeSH where internal of the tree are also embedded. This opens the possibility to experiment with different types of hierarchies, potentially identifying new modalities with corresponding hierarchies that can even increase the predictive performance.

In summary, based on our experiences, the model exhibits robust performance across the various types of hierarchies, which makes it possible to experiment with other hierarchy sources in the future, such as the GEO, protein-assay containment hierarchies for the targets, or molecular scaffolds for the drugs.

1.4. Implementation challanges

RQ: ***"What are the potential challenges in implementing these techniques in practical drug discovery pipelines?"***

AR: We appreciate your inquiry and have taken the opportunity to highlight the challenges in our revised manuscript.

The proposed approach increases the training time (see RC 2.4.). However, it is not a main concern as it is easy to implement, robust to the hyperparameters (see RC 2.3.) and the various types of hierarchies (see RQ 1.3.), moreover, the compatibility with other pairwise DTI methods makes it possible to extend with other XAI solutions. Currently, we believe the primary challenge in applying the method in practical drug discovery pipelines is the lack of large complex training data with labeled hierarchical information (see RQ 1.1) as well as the tradeoff concerning dimensionality reduction for the visualizations. We have incorporated these insights into the *Discussion and conclusions"* section of the manuscript (pages 28-30):

...
Although the method is easy to implement, robust to the hyperparameters, and does not introduce significant constraints regarding the model architecture, it increases complexity and training time.
...
Besides the new opportunities, visualizing the latent space raises new challenges when applied in practical drug discovery pipelines. A key concern requiring further investigation is the tradeoff between losing interpretability with dimensionality reduction and losing expressive power with a small latent dimension.
...
Another ~~...~~challenge to be addressed is the limited availability of public data containing abundant, labeled hierarchies, leading to the need for diverse DTI benchmarks and datasets focusing on biological hierarchies.

1.5. Related methods in the medical domain

RC: ***"The article mainly discusses biological network and medical domains. To reflect the frontier and integrity of related work, it is recommended to introduce related methods. [1] KGNN: Harnessing Kernel-based Networks for Semi-supervised Graph Classification. WSDm 2022 [2] Building Conversational Diagnosis Systems for Fine-grained Diseases using Few Annotated Data. ICONIP 2022"***

AR: We are grateful for drawing our attention to these related methods.

We have added the suggested references to the manuscript. The corresponding modifications can be found in the *Preliminaries/Drug-target interaction prediction* subsection (page 5):

The advancement of artificial intelligence technologies has significantly impacted various aspects of the medical domain, including processing biological networks [10], diagnosing patients [11], and even accelerating differentstages of drug development.

2. Reviewer #2

2.1. Case study on hierarchical information captured by hyperbolic embeddings

RC: ***"The author claim that they want to capture the hierarchical information by using hyperbolic embedding. However, they didn't provide explanation cases in that way. I suggest the author to show a case study to enhance its explainability."***

AR: We appreciate the reviewer's feedback and partially agree with it.

In our initial manuscript, we already provided two use cases in Figures 4 and 5, demonstrating the visualization of hierarchy trees and the interconnection of hierarchies from different modalities. Nevertheless, we acknowledge that the visualizations could also be performed in Euclidean space. To address this concern, we extended our analysis with an additional case study utilizing the translation operator to fully exploit the capabilities of the Poincaré visualization. In the revised manuscript, first, we introduced the translation operator in the *Preliminaries/Hyperbolic geometry* subsection (page 9), and then we incorporated a new figure (Fig 6.) and corresponding paragraph in the *Results/Latent space analysis* subsection (pages 26-27):

> While the Lorentz model has its stability, other models have other advantages. For example, the Poincaré model is well suited for visualization since it does not require an extra ambient dimension. More than that, it supports useful operators such as translation, i.e., rotating the Poincaré disc to move the origin to a given point while distances between vectors remain intact. As the spatial resolution is amplified near the origin, translation in hyperbolic space can also be considered a "zoom-in" method by moving the area of interest to the center [5]].

> The previous examples demonstrated the visualization of hierarchy trees and the intertwining of hierarchies of the two different modalities. Nevertheless, we have not yet fully exploited the possibilities of the Poincaré disk visualization. Fig 6 presents another use-case with the KIBA model utilizing the translation operator to navigate the hyperbolic space. The used model representations and the color of the nodes are the same as in Fig 4. Still, notably, due to the nondeterministic nature of Poincaré maps dimensionality reduction, the 2-dimensional visualizations are not precisely the same. We can see that by rotating the disk, substructures with their neighborhood can be examined more thoroughly on different hierarchy levels. This is possible because translating the internal nodes of the hierarchy tree to the origin where distances are perceived to be larger allows the observation of fine details.
>
> **Fig 6. Navigating the KIBA latent space by the translation operator on the Poincaré disk.** In the top row *(a-c)*, the kinase hierarchy tree is depicted, with nodes colored by hierarchy level, while below *(d-f)*, the jointly embedded drugs and targets are shown, colored by ATC1 and kinase groups, respectively. From left to right, the figure illustrates the translation process through different nodes in the hierarchy tree. Solid and dashed red arrows indicate translations yet to occur and those already completed. In the first column *(a, d)*, the original result of the Poincaré maps dimensionality reduction is presented. In the middle *(b, e)*, the internal node of the hierarchy tree corresponding to the *CAMK* group, i.e., the average of the representations belonging to that group, is translated to the origin. Finally, the last column *(c, f)* displays the neighborhood of the *CAMKL* family after translating the corresponding node of the tree to the center.

2.2. Algorithm pipeline plot

RC: ***"The introduction of the algorithm is not that clear. I suggest the author to add a pipeline plot to show the procedure of their algorithm."***

AR: We are grateful for the idea.

To improve the clarity of our methodology, we have added the suggested content (Fig 1.) to the manuscript in the *Introduction* section (page 4):

> An overview of the proposed method is provided in Fig 1.
>
> **Fig 1. Overview of the proposed method.** *(A)* As a preprocessing step, the DTI matrix for the positive and negative interactions, the lowest common ancestor distance matrices for the hierarchies, and the input representations are created and stored. *(B)* Then, during the training phase, b interactions, n drug and target anchors, and for each anchor, one positive and m negative samples are selected in each batch (in this example, b=3, n=1, m=2). Utilizing the distances between the model embeddings of the selected samples, the loss is calculated, and based on its gradient, the model weights are updated. *(C)* Finally, the resulting model allows the visualization of the joint-embedding DTI space, which now encodes interactions as well as prior hierarchies. After encoding input representations and subsequently applying dimensionality reduction, the hyperbolic latent space of the model can be visualized in two dimensions. The latent space might be subject to further analysis, such as navigating with the translation operator and identifying clusters of interest. For more details see the following subsections: *(A)* Datasets, *(B)* Model and objective function, *(C)* Latent space analysis.

Later, in the corresponding subsections, we referred back to the figure to help understand the described methods (pages 14, 16, and 22):

> An abstract overview of the resulting preprocessed data is found in Fig 1 A.
> ...
> Fig 1 B shows the batch selection and the training process, while Fig 2 depicts an overview of a hyperbolic pairwise model regularized with prior hierarchies.
> ...
> An overview of the dimensionality reduction and the visualization process is shown in Fig 1 C.

2.3. Sensitivity of the algorithm

RC: ***"Although they show the performance in the experiment part, they fail to show the sensitivity of the proposed algorithm. I suggest the author to add an ablation to show its robustness."***

AR: We appreciate the reviewer's suggestion regarding conducting an ablation study to demonstrate the sensitivity of our proposed algorithm. However, we respectfully disagree with the necessity of such an additional analysis.

In our experimental setup, we systematically tested all possible 2^3 combinations (based on whether to use the proposed drug hierarchy, target hierarchy, and the hyperbolic manifold) considering predictive performance, hierarchy preservation, and in the revised version, the training cost as well (See RC 2.4.). In other words, we have already investigated the contribution of each proposed component separately. Furthermore, in the revised manuscript, we also provided insights into the robustness of our approach concerning different types of biological hierarchies (See RQ 1.3.). Another aspect is the sensitivity to the hyperparameters, which we

have already investigated, and we found the model to be robust to most of them except the latent dimension. In the interest of conciseness and clarity, we opted not to include additional hyperparameters in our analysis, as we deemed them less relevant based on our experimental observations. However, we indeed forgot to highlight this insight in the previous version, so we updated the *Results* section accordingly (page 18):

> Computations were executed on a 32GB NVIDIA Tesla V100 GPU, and the models were implemented using the PyTorch framework. ~~After exploring various hyperparameter combinations, we identified an effective configuration that remained consistent across our study.~~ Notably, following hyperparameter optimization, we found that the model is robust considering most of its parameters except for the latent dimension, i.e., the size of the drug and target embeddings between which the distances are calculated. Therefore, we applied a previously identified effective configuration consistently throughout our study, focusing our detailed comparison solely on the latent dimension. The other hyperparameters did not significantly affect the results and, consequently, were not subjected to a thorough examination.

2.4. Efficiency of the algorithm

RC: ***"I suggest the author to analyze the efficiency of the proposed algorithm."***

AR: Thank you for pointing this out. Indeed, the aspect of efficiency needed further explanation in our manuscript.

To address this, we measured the mean and standard deviation of the time required to train one epoch on our 32GB NVIDIA Tesla V100 GPU, utilizing the same hyperparameters as in the comparative study, measuring the training times for each regularization scenario and manifold on both datasets. In the *Results/Comparative analysis* subsection of the revised manuscript, we provided an extra paragraph and extended Fig 3. to illustrate the cost of the proposed modifications alongside the performance metrics (pages 19-22):

> Fig 3 shows the resulting means and standard deviations for all evaluation metrics, as well as the time required to train the model for one epoch on our previously mentioned hardware setup.
> ...
> As for computational cost, not surprisingly, fitting the model on the larger KIBA dataset requires more time. Nevertheless, the same conclusion can be drawn from both datasets. The latent dimension does not significantly affect the training time, i.e., increasing the size of the hidden representations does not have an additional cost. However, utilizing non-Euclidean embeddings has a noticeable overhead, more precisely, a multiplicative cost, with training hyperbolic models for one epoch taking approximately 1.6 times as long as for the Euclidean ones. On the other hand, hierarchy regularization has rather a cumulative cost, with the extra cost of row and column regularizations independently added to the base training time. Notably, after fitting the model, the proposed modifications do not have an extra cost, as the regularization is only applied during training, and the extra exponential map required in the forward pass for the hyperbolic version does not significantly increase inference time.
>
> Based on the results, hierarchy regularization, at the cost of extra training time, contributes to forming a more meaningful embedding space without significantly impacting the predictive performance. Additionally, with more than approximately 30 available leaf nodes in the prior hierarchy tree, using a hyperbolic embedding space is highly recommended, as it enhances the quality of hierarchy preservation at only a small computational cost.
>
> **Fig 3. Cross-validation results on the *(a)* KIBA and *(b)* NURA datasets.** Models with different manifolds are placed in different columns, while the different rows display the various evaluation

metrics and the time required to train one epoch. Within each plot, four curves corresponding to four scenarios based on the applied a priori hierarchies depict mean and standard deviation values for different latent dimensions obtained through cross-validation.

Additionally, we mentioned the implications of these findings in the *Discussion and conclusions* section (page 28):

> Although the method is easy to implement, robust to the hyperparameters, and does not introduce significant constraints regarding the model architecture, it increases complexity and training time. As a rule of thumb, over approximately 30 leaf nodes to embed, we recommend using hyperbolic space with the regularization as it significantly increases the hierarchy preservation without sacrificing predictive performance.

2.5. Further polish the paper

RC: ***"Some writting could be polished. I suggest the author to further polish the paper and keep it concise."***

AR: We agree with the reviewer's assessment.

Accordingly, we have carefully revised the manuscript to correct errors and improve readability by reformulating some paragraphs. Throughout the revision process, we also aimed to address all questions and suggestions raised by the reviewers while ensuring that the manuscript remains concise and focused. For more details see all the highlighted modifications in the track changes manuscript.

2.6. Related methods in the medical domain

RC: ***"I suggest the author to add a section to introduce the current progress of XAI. I list some relevant papers as follow:***
1 Guerdan, Luke, Alex Raymond, and Hatice Gunes. "Toward affective XAI: facial affect analysis for understanding explainable human-ai interactions." In Proceedings of the IEEE/CVF International Conference on Computer Vision, pp. 3796-3805. 2021.
2 Weitz, Katharina, Dominik Schiller, Ruben Schlagowski, Tobias Huber, and Elisabeth André. "" Do you trust me?" Increasing user-trust by integrating virtual agents in explainable AI interaction design." In Proceedings of the 19th ACM International Conference on Intelligent Virtual Agents, pp. 7-9. 2019.
3 Chen, Ziheng, Fabrizio Silvestri, Jia Wang, He Zhu, Hongshik Ahn, and Gabriele Tolomei. "Relax: Reinforcement learning agent explainer for arbitrary predictive models." In Proceedings of the 31st ACM International Conference on Information & Knowledge Management, pp. 252-261. 2022.
4 Goyal, Yash, Ziyan Wu, Jan Ernst, Dhruv Batra, Devi Parikh, and Stefan Lee. "Counterfactual visual explanations." In International Conference on Machine Learning, pp. 2376-2384. PMLR, 2019.
5 Biran, Or, and Courtenay Cotton. "Explanation and justification in machine learning: A survey." In IJCAI-17 workshop on explainable AI (XAI), vol. 8, no. 1, pp. 8-13. 2017.
6 Yarkoni, Tal, and Jacob Westfall. "Choosing prediction over explanation in psychology: Lessons from machine learning." Perspectives on Psychological Science 12, no. 6 (2017): 1100-1122.
7 Parimbelli, Enea, Tommaso Mario Buonocore, Giovanna Nicora, Wojtek Michalowski, Szymon Wilk, and Riccardo Bellazzi. "Why did AI get this one wrong?—Tree-based explanations of machine learning model predictions." Artificial Intelligence in Medicine 135 (2023): 102471.
8 Lundberg, Scott M., Gabriel Erion, Hugh Chen, Alex DeGrave, Jordan M. Prutkin, Bala Nair, Ronit Katz, Jonathan Himmelfarb, Nisha Bansal, and Su-In Lee. "From local explanations to global understanding with explainable AI for trees." Nature machine intelligence 2, no. 1 (2020): 56-67."

AR: We appreciate the reviewer's insight and the provided list of relevant papers on XAI.

As suggested by the reviewer, we moved all previous XAI parts from the *Introduction* section and the *Preliminaries/Drug-target interaction prediction* subsection to a newly created *Preliminaries/Explainable AI* subsection. Besides the previously existing paragraphs, we extended the manuscript with a more thorough introduction to XAI, including most of the listed papers that we could access, as well as other papers we found relevant. The resulting dedicated subsection takes the following form (pages 6-7):

Explainable AI

Despite their success, machine learning models are often perceived as black boxes, hindering their interpretability, a critical factor, particularly in medical applications [19]. Therefore, the need for *eXplainable Artificial Intelligence* (XAI) solutions is increasing, aiming to provide transparency and facilitate decision-making. This need has led to the development of various XAI methods, ranging from model-specific to more general, known as model-agnostic approaches [20]. Besides providing interpretable models for machine learning practitioners, there is also a growing demand to increase user trust [21] and even understand how users engage with the explanations [22].

A widespread approach in the medical domain is utilizing interpretable tree-based predictive architectures [19,23]. Besides, there are existing solutions to enhance the explainability of more complex models, such as deep neural networks, for instance, by generating counterfactual explanations [24,25] or by attributing the predictions to the input features [26].

XAI approaches are frequently used in drug development to enhance model transparency, identify crucial input features, validate output acceptability, offer insights to decision-makers, and estimate the uncertainty associated with predictions [27]. A prominent example is the utilization of the *SHapley Additive exPlanations* (SHAP) methodology to provide local explanations by identifying compound features responsible for kinase inhibitor activity prediction [28,29]. Besides, pairwise DTI predictors can also be subjected to XAI solutions, with most recent methods relying on the interpretable-by-design attention mechanism. For instance, some solutions leverage mutual learning attention for drug and target encoders coupled with attention visualization for local interpretation and identification of relevant input features [30]. Others use cross-modal attention with regularization guided by non-covalent interactions [31], and some graph-based models identify protein binding sites for enhanced interpretability [32].

In our current ~~research, we continue to work with joint-embedding~~ paper, we also use pairwise DTI models. However, ~~this time, we explore how~~ instead of local input interpretations provided by the attention mechanism, we aim to make the joint-embedding latent space more explainable by incorporating *a priori* drug and target hierarchies~~can be embedded in the shared latent space~~. Besides evaluating the effect of ~~integrating prior~~ embedding prior biological hierarchies, we extend our investigation to non-Euclidean ~~embeddings~~representations, considering their potential advantages in preserving ~~hierarchies~~hierarchy trees.
